# Brain Functional Connectivity During First- and Third-Person Visual Imagery

**DOI:** 10.3390/vision9020030

**Published:** 2025-04-06

**Authors:** Ekaterina Pechenkova, Mary Rachinskaya, Varvara Vasilenko, Olesya Blazhenkova, Elena Mershina

**Affiliations:** 1 Laboratory for Cognitive Research, School of Psychology, Faculty of Social Sciences, HSE University, 101000 Moscow, Russiavvasil5664@gmail.com (V.V.);; 2 Institute of Social Sciences, Russian Presidential Academy of National Economy and Public Administration, 119571 Moscow, Russia; 3 Faculty of Arts and Social Sciences, Sabanci University, 34956 Istanbul, Turkey; olesya@sabanciuniv.edu; 4 University Clinic, Lomonosov Moscow State University, 119991 Moscow, Russia

**Keywords:** mental imagery, visual imagery, perspective, vantage point, first-person perspective, third-person perspective, fMRI, functional connectivity

## Abstract

The ability to adopt different perspectives, or vantage points, is fundamental to human cognition, affecting reasoning, memory, and imagery. While the first-person perspective allows individuals to experience a scene through their own eyes, the third-person perspective involves an external viewpoint, which is thought to demand greater cognitive effort and different neural processing. Despite the frequent use of perspective switching across various contexts, including modern media and in therapeutic settings, the neural mechanisms differentiating these two perspectives in visual imagery remain largely underexplored. In an exploratory fMRI study, we compared both activation and task-based functional connectivity underlying first-person and third-person perspective taking in the same 26 participants performing two spatial egocentric imagery tasks, namely imaginary tennis and house navigation. No significant differences in activation emerged between the first-person and third-person conditions. The network-based statistics analysis revealed a small subnetwork of the early visual and posterior temporal areas that manifested stronger functional connectivity during the first-person perspective, suggesting a closer sensory recruitment loop, or, in different terms, a loop between long-term memory and the “visual buffer” circuits. The absence of a strong neural distinction between the first-person and third-person perspectives suggests that third-person imagery may not fully decenter individuals from the scene, as is often assumed.

## 1. Introduction

### 1.1. Perspective Taking, and First-Person Versus Third-Person Perspectives

Vantage point, or perspective, refers to the position from which an individual views or mentally represents a scene. People can adopt different vantage points when encoding, retrieving and using spatial information [1]. This ability may be fundamental to the development of human thinking, as it supports mental object manipulation, understanding others’ beliefs, and reflexive reasoning. Adult individuals can mentally shift their imagined perspectives depending on the demands of a task. The ability to shift away from an egocentric viewpoint and to consider multiple aspects of a situation emerges in the course of a child’s cognitive development and was described by Jean Piaget as decentration [2]. Besides reasoning, decentration and perspective taking are intensively involved in memory and imagination. The ability to shift between personal and external perspectives influences how events are recalled and communicated.

Autobiographical memory research distinguishes between first-person memories (often called field-perspective in the memory literature), where events are recalled as if re-experiencing them through one’s own eyes, and third-person (observer) memories, where individuals recall events as if watching themselves from an external vantage point [3]. First-person recall is often associated with a stronger sensory and emotional reliving of the event. Third-person recall tends to be used in self-distancing strategies, reducing emotional intensity and promoting reflection [4].

During imagery, individuals also can adopt either a first-person (representing space from their own viewpoint) or third-person (external observer) perspective when visualizing scenes or events [5]. Notably, besides decentering/detaching from the first-person vantage point, third-person perspective imagery allows visualizing one’s own body in a mental image constructed from an external viewpoint.

Imagining a scene from a third-person perspective should require more mental effort compared to a first-person perspective in both of the following respects: first, additional cognitive load may be needed to reconstruct the scene from an unfamiliar viewpoint [6]; second, integrating one’s own body position with a scene viewed externally may also demand extra resources, as suggested by an electrophysiological study showing more pronounced neural correlates of cognitive effort in the frontal lobes in third-person compared to first-person imagery [7].

Third-person imagery may introduce more spatial distortions compared to first-person imagery. Since we naturally experience the world from our own eyes, projecting ourselves into an external viewpoint can lead to miscalculations in spatial relationships [8]. Additionally, subjective body ownership, a sense of embodiment, and reactions to threat are stronger during the first-person perspective than the third-person perspective [9]. Visuomotor information is more effectively conveyed through a first-person perspective versus a third-person perspective. Also, latency to imitate an action shown in video-clips is shorter during the first-person perspective compared to the third-person perspective [10,11].

Notably, the first-person vs. third-person distinction is different from the concept of allocentric and egocentric coordinate systems. The egocentric coordinate system defines locations relative to the observer’s body (e.g., left-right, near-far). This system is closely linked to action-oriented processing and self-movement [12]. The allocentric coordinate system defines locations relative to external objects or landmarks, independent of the observer’s current position [13]. While allocentric and egocentric spatial processing are governed by distinct cognitive and neurological processes [14,15], they also interact dynamically in many spatial tasks. Perspective-taking tasks, as well as most real-world navigation, way-finding, and spatial orientation, require egocentric processing since they involve maintaining and updating self-referenced spatial relationships. Additionally, even though common perspective-taking tasks [16] include a map, which provides a global (allocentric) layout, study participants must simulate an embodied presence within the map, making the task predominantly egocentric. If the task required participants to view the spatial layout from an external, bird’s-eye perspective and indicate directions between objects independently of their own position, it would be more allocentric. However, when imagining oneself from the outside, a person is more likely to simply change one egocentric coordinate system to another, moving its origin to the location of another subject (imagined embodied human observer), than to switch to an allocentric system [17].

Modern visual media (e.g., video games, movies, virtual reality) frequently present experiences from a third-person perspective or allow users to switch between perspectives (e.g., navigators in vehicles). This might make the practice of remembering and imagining oneself from the outside (a third-person perspective) more common. From a neuroscience perspective, it raises an interesting question about what these practices might change in the human brain. Given the importance of decentration for the development of human thought, surprisingly little is known about how different mental vantage points are represented in the brain during internally generated experiences. Revealing the underlying mechanisms of imagery perspectives may not only advance theoretical knowledge but also promote education (e.g., [18]), immersive media [19], and psychotherapy [20].

### 1.2. Brain Underpinnings of First-Person and Third-Person Perspectives

We are aware of only one published study directly comparing the neural correlates of the same imagery tasks performed from the first- and third-person perspectives, which is a PET study by Ruby and Decety [21]. When the participants were performing imagery tasks from the third-person perspective, more pronounced activation was recorded in the medial prefrontal cortex, the precuneus, and the lateral parietal cortex, which are now known to belong to the default-mode network (DMN). In an early unpublished fMRI study [17], participants were asked to count objects seen from their own perspective and from the perspective of an avatar; “externalizing” the perspective was accompanied by activation in the right lateral parietal cortex, and counting from one’s own perspective was accompanied by activation in the medial parietal and prefrontal cortex. Meanwhile, Tomasino et al. [22] found no difference in activation when asking participants to imagine situations described in first- and third-person sentences.

An ERP study by Arzy et al. [23] showed that, in healthy people, embodied imaginary self-location is associated with the greater activity in the extrastriate body area (EBA), and disembodied imaginary self-location is associated with greater activity in the temporoparietal junction (TPJ). Another study [24] demonstrated that transcranial magnetic stimulation over the TPJ may disrupt the imagery process when healthy participants try to imagine an out-of-body experience. However, an fMRI study of a clinical case involving a participant who could voluntarily have an out-of-body experience showed that, compared to imagining herself from the outside, the out-of-body experience was accompanied by increased activation in the lingual gyrus (visual cortex) and decreased activation in the orbitofrontal cortex, but it did not involve any greater activation of the TPJ [25].

Several more recent neurocognitive studies were conducted in the field of autobiographical memory. A resting-state fMRI study has shown that seeing one’s own body during episodic encoding modulates the subsequent resting-state functional connectivity between the right hippocampal formation and the neocortex [26]. In one direct task-based fMRI comparison, the observer perspective was associated with greater activity in the right precuneus and in the right TPJ [27], while in another study, observer memories were accompanied by significantly decreased activity of the insulae and the left somato-motor cortex compared to field memories [28]. Based on these and some other relevant findings, a neurocognitive model suggested by St. Jacques [29] incorporates two brain subsystems supporting the visual perspective during episodic memory retrieval: the bodily self subsystem, including the somatosensory cortex, the insular cortex, and the right TPJ; and the viewpoint-specific mental imagery subsystem, including the angular gyrus and the precuneus.

To summarize, the available neuroimaging data indicate a wide range of potential brain mechanisms for externalizing the perspective during imagery. However, none of the available studies have directly compared the task-based functional connectivity (FC) associated with first-person and third-person perspectives during mental visual imagery in the brain. To obtain more straightforward evidence related to the difference in the brain underpinnings of first-person and third-person self-imagery, we conducted an experiment in which the same participants were asked to perform visual and motor imagery tasks taking two perspectives—the first-person and the third-person—during fMRI scanning. In the two imagery conditions, we compared whole-brain activation and task-based FC.

We used two fMRI paradigms developed by the Coma Science Group and widely used as imagery tasks in healthy volunteers and in clinical populations: imaginary tennis and imaginary navigation around one’s house [30]. Comparing the neural correlates of the vantage points across the two tasks allows for disentangling the invariant effects of perspective from the task-specific mechanisms. The fact that some previous neuroimaging results indicate the involvement of components of the DMN, which is sensitive to task difficulty, together with other findings indicating that one perspective may take greater mental effort than another [7], we asked our participants to report at the end of the experiment which of the two perspectives they considered more difficult.

While we expected possible greater signs of embodied cognition in the first-person perspective (stronger activity or FC in the bodily self subsystem, according to St. Jacques [29]), given the heterogeneous results of the previous few fMRI studies, we applied an exploratory approach and looked for any potential task-based activation differences in FC.

## 2. Materials and Methods

### 2.1. Participants

Twenty-eight healthy volunteers from the academic community of Moscow took part in the study. Inclusion criteria were age 18–45 y.o. and Russian being the native language; exclusion criteria were self-reported history of neurological or psychiatric disease, hearing problems, and contraindications to MRI. All participants passed the MRI safety screening and provided written informed consent before the experiment. Handedness was assessed with the Edinburgh Handedness Inventory by Oldfield [31].

Data from two participants were excluded from the analysis (one male, one female; one for not following the instructions and opening their eyes during the scanning session, and another for not hearing the task-switching commands due to technical reasons), resulting in 26 participants in the final sample (18 females, 8 males; mean age 24 ± 6 y.o.). Their mean laterality quotient (LQ10) was 75 ± 21, and all participants were right-handed with the exception of two who were ambidextrous (0 < LQ10 < 40).

### 2.2. Procedure

Participants were not trained to perform the tasks, but they received detailed instructions before the scanning. Two imagery tasks, tennis and house navigation, were explained. For the tennis task, the experimenter asked the participant to imagine being on a tennis court, serving, and returning subsequent shots in a game with an imaginary opponent. If the participants had no personal experience of playing tennis, they were asked to imagine badminton, which is a more popular outdoor activity in Russia. The participants were asked to start imagining the game at the ‘Play tennis’ command and to keep the game unraveling in their mind until the ‘Stop’ command. For the house navigation task, the experimenter asked the participant to imagine wandering across a familiar house or apartment with several rooms (preferably their own or family home) and visualizing the furnishings of these rooms and the objects in them along the way. The participants were asked to start navigating at the ‘Walk around the house’ command and to keep up this mental activity until the ‘Stop’ command.

**Figure 1:**
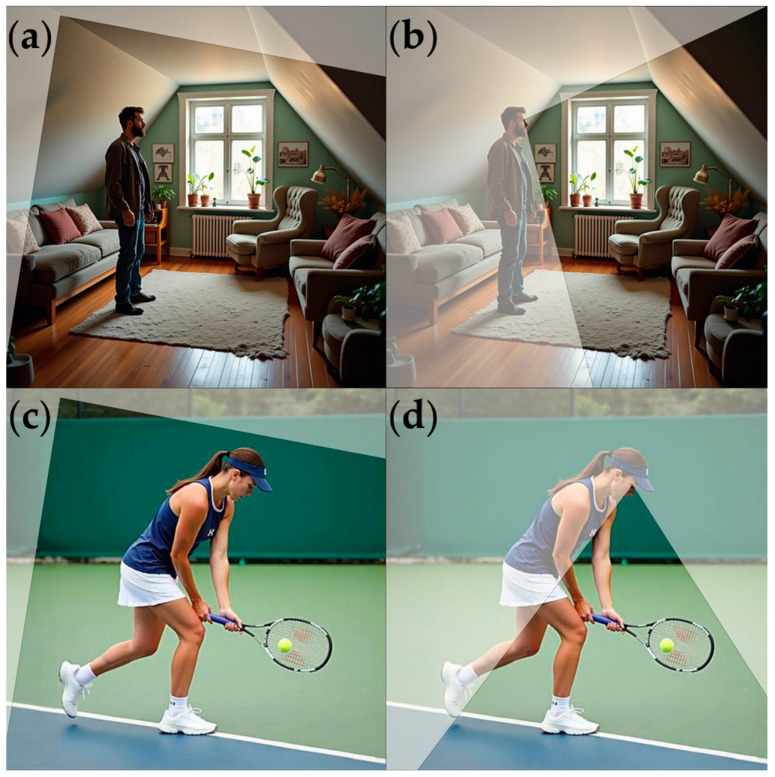
A visual illustration of the scenes implied in the imagery tasks: (**a**) the house navigation task with third-person perspective; (**b**) the house navigation task with first-person perspective; (**c**) the tennis task with third-person perspective; (**d**) the tennis task with first-person perspective. The pictures were generated using FLUX Tools by Black Forest Labs (https://blackforestlabs.ai/ (accessed on 1 March 2025)).

Then, the difference between the first-person and the third-person perspectives was explained. Participants were instructed to look at the imaginary activities either from the vantage point of their own eyes or to look at themselves as seen by an outside observer, as if they were watching a movie about themselves. A conditional visual illustration of these scenes is presented in Figure 1.

In the scanner, all tasks were performed with eyes closed, and the experimenter only asked the participants to open their eyes in between the functional sessions to prevent them from falling asleep.

**Figure 2:**
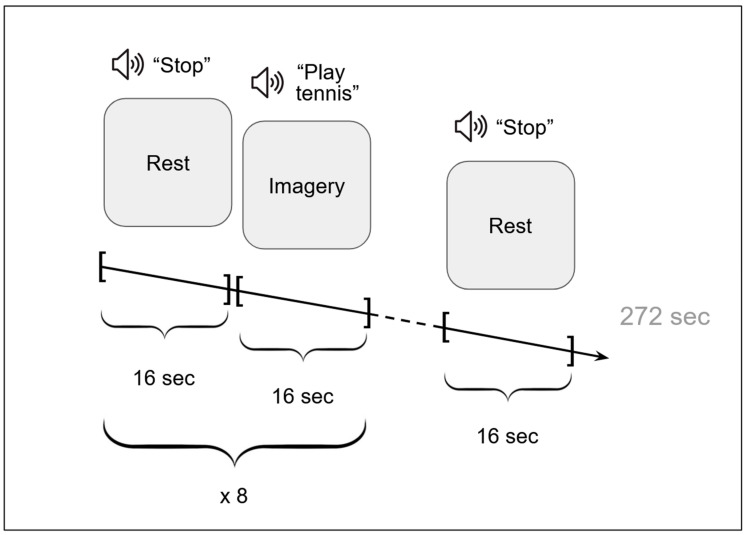
The order of task and rest blocks in the ‘Play tennis’ session.

For each participant, four sessions of functional imaging were performed, one per combination of the imagery task (tennis or house navigation) and viewpoint (first or third perspective). The order of the four conditions (sessions) was counterbalanced across participants using a Latin square design. Participants were informed of the upcoming task and the required mental viewpoint before the start of each session. Within each session, the task was organized in 16 s blocks alternating with 16 s resting periods (rest, or the baseline condition, lasted from the ‘Stop’ command to the next imagery command). The order of blocks in sessions is illustrated in Figure 2. There were 8 task blocks and 9 resting blocks in each session, which is a typical session length for the Coma Science Group paradigms (e.g., see [32] suggesting comparable results for 5 to 10 blocks for the 1.5T and 5 blocks for 3T scanners).

At the end of the experiment, the participants were asked which of the two perspectives was more difficult for them to imagine.

### 2.3. Functional MRI Data Acquisition and Analysis

#### 2.3.1. Functional MRI Data Acquisition

MRI data were acquired with a 1.5T Magnetom Avanto scanner (Siemens, Erlangen, Germany) located at the Medical Research and Educational Centre, Lomonosov Moscow State University in Moscow, Russia, which is equipped with a standard Head Matrix 12-channel coil. During the scanning sessions, instructions to participants were provided via SensaVue (InVivo Corporation, Gainesville, FL, USA).

In each of the four functional sessions, 140 T2*-weighted functional images were acquired, and 4 initial volumes in each session were discarded. Sessions lasted for 4 min 46 sec each. The following parameters were specified for the gradient-echo echo-planar imaging (GRE EPI) pulse sequence: TR/TE/FA = 2000 ms/50 ms/83°, FoV = 256 mm × 256 mm × 96 mm, matrix size = 64 × 64 × 22, slice gap 5%, resulting in an isotropic voxel size of 4 mm. Each volume covered the whole brain with slices oriented parallel to the AC/PC line. For some participants, the total coverage of the brain volume with the specified FoV was not possible, so the inferior portions of the cerebellum were not imaged. Field maps with the same slice prescriptions as the functional images were acquired at the midpoint of the experiment with a standard Siemens double-echo GRE field mapping sequence (TR/TE1/TE2 = 460 ms/4.76 ms/9.52 ms). T1-weighted MPR structural images with 1 mm isotropic resolution were also acquired for each participant.

#### 2.3.2. Functional MRI Data Analysis: Activation

Data were preprocessed with SPM 12 v. 7771 (Wellcome Institute of Cognitive Neurology, www.fil.ion.ucl.ac.uk/spm (accessed on 1 March 2025)) and the following steps: slice timing correction; realignment and fieldmap-based unwarping; spatial co-registration of the structural and functional images; segmentation of the average structural volume into six tissue volumes; normalization into Montreal Neurological Institute (MNI) space; and spatial smoothing of the functional images with a Gaussian kernel of 8 mm full width at half-maximum (FWHM). Six residual head motion parameters (three for translation and three for rotation) were extracted during the realignment step.

To reveal the task-based activation, for each participant, the MR signal in each voxel was modeled using the general linear model with canonical hemodynamic response function. The data were analyzed as a block design with one experimental condition (imagery task) per session, while the baseline condition (rest) was not explicitly modeled to avoid model redundancy. Six parameters describing head motion throughout the experimental session were included into the model as nuisance regressors. *T*-test contrasts for the BOLD signal change evoked by imagery were obtained for each session (i.e., task × perspective combination). Four contrast images per individual (one per condition) entered a flexible-factorial second-level SPM model with a subject factor introduced to control for repeated measures. First, the group activation maps were constructed for all imagery conditions vs. baseline (rest) to reveal brain areas involved in closed-eye visual imagery in general. Next, the between-task contrasts were assessed across both perspectives to reveal the neural correlates specific to each imagery task (tennis and house navigation). Finally, the between-perspective contrasts were estimated across both tasks and within each task individually. The results were assessed with a topological FDR correction for multiple comparisons, with a cluster-wise threshold of *p*_FDR_ < 0.05 (q = 0.05) based on a voxel-wise threshold of *p* = 0.001 uncorrected. Peak activation coordinates were labeled with the Harvard–Oxford maximal likelihood cortical and subcortical atlases in the version implemented in Conn [33].

#### 2.3.3. Functional MRI Data Analysis: Functional Connectivity

CONN Functional Connectivity Toolbox v. 22a was used for the task-based functional connectivity statistical analysis. First, the data initially preprocessed with SPM12 for the activation analysis entered several standard denoising Conn procedures [34]. In order to account for the residual motion-induced artifacts and physiological noise, head motion artifact detection was performed with the Artifact Detection Toolbox (ART; [35]) at medium-level thresholds; outliers were identified as images demonstrating scan-to-scan head motion of more than 0.9 mm or global mean intensity change of more than 5 SDs. Next, an anatomical component-based noise correction technique (aCompCor; [36]) was applied; noise ROI were defined within the individual CSF and white matter masks for each participant. Head motion parameters, outliers, CSF/white matter signal, main BOLD-signal effects of the imagery task and rest blocks, and a linear detrending term were included into the denoising model and regressed out. Finally, a standard temporal high-pass filter with a cutoff of 0.008 Hz was applied in order to restrict the analysis to the frequency band which is characteristic of the task-based fMRI BOLD signal.

Two exploratory approaches to the task-based functional connectivity analysis were taken: voxel-to-voxel and entire atlas ROI-to-ROI. The Harvard–Oxford atlas in the version implemented in the Conn toolbox (105 labels) was used for the ROI definition and results labeling.

For a voxel-to-voxel analysis, the intrinsic connectivity contrast (ICC) values were computed for each voxel in the whole brain. ICC was obtained as a mean absolute value of the correlations of the time series for a given voxel with all other voxels included in the analysis [37]. As the ICC coefficients were further used for within-subject comparisons, the across-brain normalization was not applied.

For both exploratory analyses, three contrasts were estimated: the first-person vs. third-person perspectives were compared across both tasks and within each task individually. The mean-centered percent of the valid scans per subject was included into the model as a between-subject covariate in order to account for possible task-related individual differences in residual participant head motion.

Differences in ICC were assessed with both parametric and non-parametric statistics for cluster-based inference with an FDR correction for multiple comparisons [38,39]. Differences in ROI-to-ROI connectivity were assessed with a non-parametric network-based statistics (NBS) analysis [40]. NBS provides an analog to the cluster-based inference for the ROI-to-ROI analyses, i.e., a set of statistical techniques for testing hypotheses about the clusters of connections (networks) rather than individual connections. The multiple comparison correction in NBS is implemented similarly to topological (cluster-wise) family-wise error/false discovery rate (FWE/FDR) corrections for the voxel-based maps. For the current study, we used NBS by intensity (mass) statistics with a connection-level threshold of *p* < 0.001 uncorrected and a cluster-level threshold of *p* < 0.05, FDR-corrected at the network level, and looked for any network (set of clustered connections) that showed a significant change in connectivity between the first-person and third-person perspective in both imagery tasks or in each task separately.

## 3. Results

### 3.1. Subjective Reports

Among 26 participants, 10 reported that the first-person condition was less difficult, 7 experienced it as more difficult, 3 people reported that the first-person perspective required less mental effort for the house navigation task and more effort for the tennis task, and 6 people reported both imagery vantage points as being of equal difficulty.

### 3.2. Brain Activation

**Figure 3:**
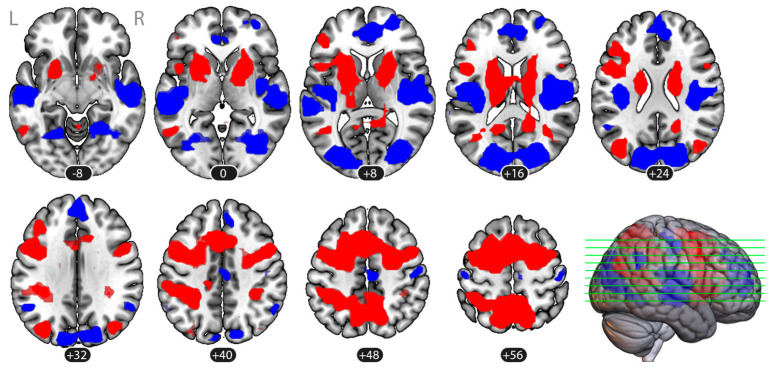
Overall neural correlates of closed-eye imagery tasks compared to baseline (rest): group map, *n* = 26, voxel-level threshold *p* < 0.001, cluster-wise threshold *p*_FDR_ < 0.05. Activation (imagery > baseline) is shown in red; deactivation (baseline > imagery) appears in blue. The statistical map is overlaid onto an MNI template. All data are presented in the MNI space.

Figure 3 presents the overall activation map of areas manifesting significantly greater or significantly less BOLD signal in both imagery tasks and in both perspectives compared with the baseline (rest), with relevant cluster-wise statistics shown in Table 1. Regions that were more activated in imagery vs. baseline included bilateral frontal and parietal areas typically assigned to the frontoparietal control network (middle and superior frontal gyri, supplementary motor area, superior parietal lobule), the bilateral occipitoparietal areas, and the basal ganglia. Regions more activated in baseline vs. imagery included the bilateral operculum, auditory cortex, occipital cortex, angular gyrus, postcentral gyrus, posterior cingulate, and medial prefrontal cortex.

**Figure 4:**
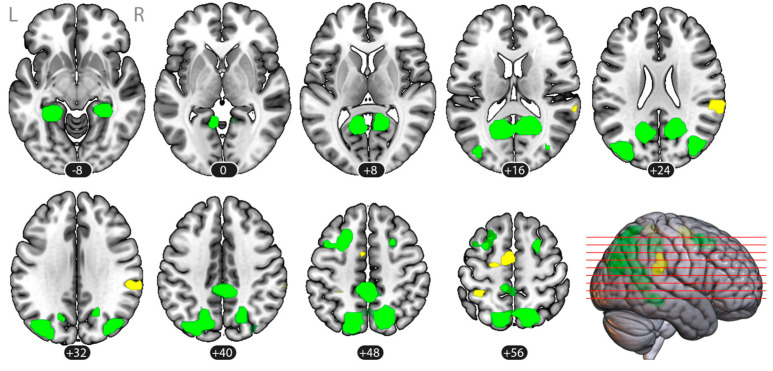
Specific neural correlates of the two closed-eye imagery tasks compared to each other: group map, *n* = 26, voxel-level threshold *p* < 0.001, cluster-wise threshold *p*_FDR_ < 0.05. The tennis > house navigation contrast is shown in yellow, the house navigation > tennis contrast in green. The statistical map is overlaid onto an MNI template. All data are presented in the MNI space.

The between-task contrasts are shown in Figure 4 and Table 2. As demonstrated by the group activation map, the imaginary tennis task elicited greater activation compared to the imaginary house navigation task in the supplementary motor area (SMA), the right supramarginal gyrus, and the left superior parietal cortex. In turn, the imaginary house navigation task resulted in greater engagement of the bilateral hippocampal gyrus, the occipitotemporal cortex, and the frontal eye field regions, compared to the imaginary tennis task.

Finally, the analysis revealed no significant differences in activation elicited by the first-person and the third-person vantage points in either of the imagery tasks, nor in both tasks analyzed together.

### 3.3. Task-Based Functional Connectivity

#### 3.3.1. Voxel-to-Voxel Connectivity: Intrinsic Connectivity Contrast

Neither parametric nor nonparametric statistics revealed any significant differences in whole-brain ICC between the first-person and third-person vantage points in either of the imagery tasks, nor in both tasks considered together.

#### 3.3.2. ROI-to-ROI Connectivity

**Figure 5:**
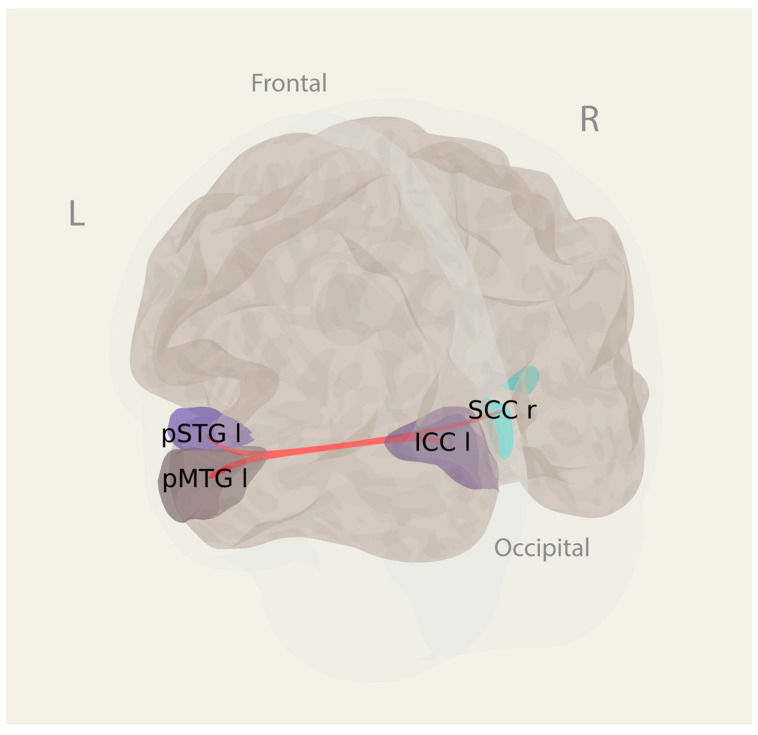
A network identified by the NBS connectivity analysis (mass/intensity) with an FDR correction for multiple comparisons and demonstrating greater functional connectivity in the first-person vantage point in visual imagery, compared to the third-person vantage point. ICC l: the left intracalcarine cortex; SCC r: the right supracalcarine cortex; pSTG l: the posterior division of the left superior temporal gyrus; pMTG l: the posterior division of the left middle temporal gyrus.

The whole-brain atlas-wide exploratory ROI-to-ROI analysis implicated 5460 connections among 105 ROIs. For the imaginary tennis and imaginary house navigation tasks, considered individually, the NBS analysis found no differences between the first-person and third-person vantage points. However, for both tasks taken together, the NBS analysis identified a network of four ROIs and three connections showing greater functional connectivity during the first-person imagery compared to the third-person imagery, regardless of the specific task (Mass = 108.87, *p*_FDR_ = 0.045). The ROIs included the left intracalcarine cortex (ICCl), the right supracalcarine cortex (SCCr), the posterior division of the left superior temporal gyrus (pSTGl), and the posterior division of the left middle temporal gyrus (pMTGl). The details of the connections are presented in Figure 5 and Table 3.

## 4. Discussion

### 4.1. Brain Activity and Connectivity Specific for the First-Person and Third-Person Perspectives in Imagery

The present study has demonstrated that different perspectives while performing mental imagery tasks such as imaginary tennis and house navigation result in an unexpectedly subtle difference in brain activity and connectivity. This is especially surprising since perspective taking seems to be a fundamental ability that underlies human thinking.

The two closed-eye imagery tasks used in our study elicited activation in the frontoparietal control network, typical of voluntary visual mental imagery, as suggested by a recent meta-analysis [41]. In line with the meta-analysis results, our data did not reveal any significant group-level activation in the early visual cortex during the closed-eye imagery tasks. On the contrary, some portions of the early visual and auditory cortices, along with the DMN components, were more activated during the baseline (resting) conditions compared to visual mental imagery, which is consistent with the idea that the early visual cortex may be involved in mental imagery through inhibitory connections [42]. The revealed task-specific activation also aligns with the task demands and the previous literature: the imaginary tennis task demonstrated greater engagement of SMA, while the imaginary navigation task demonstrated greater engagement of the parahippocampal place area (PPA; e.g., [30]).

No significant differences were found in activation elicited by the first-person and third-person conditions. This lack of difference may support the idea that at least some previous finding associating perspective taking with functioning of the DMN system (e.g., [21]) may be due to an imbalance in the difficulty of first-person and third-person perspective taking. However, the third-person perspective that demonstrated greater recruitment of the precuneus in the study by Ruby and Decety is generally associated with greater mental effort (e.g., [6,7]), while the DMN is usually more activated under the less difficult condition. Our results suggest that the ratings of the relative subjective difficulty of the two imaginary vantage points may substantially vary across individuals: more than half of our participants reported either the third-person perspective to be easier to take at least in one task, or both perspectives being equally difficult.

Such a proportion may indicate the effect of modern visual media (e.g., video games, movies, virtual reality), which frequently present experiences from a third-person perspective. Exposure to these forms of visualization may have trained individuals to adopt external perspectives, reducing the perceived cognitive effort. This increased exposure may facilitate greater flexibility in adopting different vantage points. Although such an assumption needs further empirical testing, existing evidence suggests that virtual reality facilitates seamless transitions between first- and third-person perspectives, and that users perceive perspective not as a strict dichotomy but as a continuum of experience [43]. This adaptability may reduce the cognitive cost traditionally associated with switching between perspectives [7,44], thereby minimizing the expected differences in neural and behavioral measures.

Guterstam [45] demonstrated that, under specific conditions, healthy individuals can experience both dual body ownership and dual self-location simultaneously. A similar experience may occur when participants simultaneously imagine themselves being inside the scene from a first-person perspective while also observing it from an external, third-person perspective; such simultaneous co-existence of vantage points in imagery has been hypothesized for sports imagery [46]. In the same vein, our instruction did not fully remove the participants from the imaginary scene, but rather combined the first-person view with an additional third-person perspective. The participants might still be mentally anchored in an egocentric way, merely observing from a shifted viewpoint, rather than truly stepping outside themselves. This would make third-person and first-person imagery more similar than expected, thus minimizing the differences between the recruited neural substrates.

The lack of difference between the vantage points could indicate a lack of full decentration in other tasks similar to ours, where participants are instructed to observe themselves while remaining inside the scene. This finding is particularly relevant to therapeutic approaches for post-traumatic stress disorder (PTSD), which often employ third-person perspective techniques as a way to reshape subsequent memories and to reduce emotional intensity through decentration [29]. However, our results suggest that simply instructing individuals to observe themselves from an external viewpoint may not be sufficient to fully detach them from the scene.

At the same time, our findings did not provide support for the idea that the first-person perspective loads the bodily self system more than the third-person perspective does, as we initially hypothesized. A possible explanation may arise from the fact that the model by St. Jacques (2019) [29] is based on autobiographical memory research, while our study addressed mental imagery. According to a two-dimensional theory of visual perspective, following Nigro and Neisser’s original definition of the third-person perspective [3], first-person vantage point predominance is only one dimension of mental visual perspective, while the other is third-person self-visibility. Due to this two-dimensional nature, first-person and third-person perspectives may interact with the type of representational process, and therefore, the same mental vantage point would have different effects in terms of affect-ladenness, motivation, and vividness on more recent vs. older autobiographical memories, mental imagery, and episodic future thinking [47].

It is noteworthy that the only alteration of the neural correlates of visual mental imagery caused by a change in the vantage point in our study was found in the functional connectivity domain rather than activation, thus indicating that perspective taking may reshape the current processes within the mental imagery network rather than recruiting additional substrates. The first-person perspective is characterized by greater FC in the subnetwork connecting the early visual cortex with the posterior middle and superior temporal gyri in the left hemisphere. The posterior middle temporal cortex is implicated in controlled retrieval from the semantic memory [48], and therefore, the observed functional connectivity change might indicate that the first-person perspective features a closer sensory recruitment loop (in terms of working memory theory, see Phylactou et al., 2022 for a recent review [49]), or a loop between the “visual buffer” and the contents of long-term memory in terms of the quasi-pictorial theory of mental imagery [5,50]. Along with the fact that some parts of the early visual cortex in our study were deactivated during the closed-eye visual imagery tasks compared to baseline (rest), the observed FC change in its other parts may indicate that the functioning of the above-mentioned loop involves a more complex and nuanced mechanism than simply activation of the entire early visual cortex during mental image construction.

### 4.2. Study Limitations

Our study had several limitations. First, the Coma Science Group imagery tasks that we used do not implement any form of control of the imagery content; therefore, we have no means to ensure that the participants followed the task instructions other than their reports and anecdotal comments, e.g., complaining of not having enough space in their house to step out of their imaginary self. One direction of future research may be the development of a more controlled task that would allow the experimenters to verify the vantage-point aspect of the imager’s internal experience.

Second, we used only two visual-motor imagery tasks, which constrains the possible generalization of our results.

Finally, our study may be underpowered due to its exploratory nature. We were unable to perform the power analysis and estimate the required sample size due to the lack of previous studies addressing the same effects. Not even regions of interest for potential effects were known to us in advance. Therefore, we collected a sample typical for this field of study (cf. the sample sizes reported in papers selected for the Spagna et al.) meta-analysis, which rarely exceed 20 participants [41]).

## 5. Conclusions

The present work shows that, despite the great importance of visual perspective taking for the development of human cognition, the difference in the brain mechanisms underlying the first-person and the third-person perspective in visual mental imagery may be subtle and is seen in the functional connectivity domain rather than activation. This fact indicates that the perspective taking modulates the functioning of the mental imagery network rather than recruiting different substrates. The observed alterations affected a small subnetwork, including some portions of the early visual cortex and the left posterior temporal areas, which manifested stronger functional connectivity during first-person perspective imagery. This finding suggests a closer sensory recruitment loop, or, in terms of the quasi-pictorial theory of mental imagery, a loop between long-term memory and the “visual buffer”, for the first-person mental vantage point.

Future research should explore possible ways to better isolate third-person perspective effects as well as the effects of the two aspects of perspective taking: the ability to decenter/detach the vantage point and the ability to visualize one’s own body in a mental image constructed from an external viewpoint, each of which may have their own neural correlates.

## Figures and Tables

**Table 1:** Clusters of activation characteristic to both imagery tasks and perspectives in all participants.

Cluster	Volume, Voxels (mm^3^)	*p*_FDR_	MNI Coordinates (Center of Mass)			Region Labels ^1^
			x	y	z	
Imagery > Rest (Baseline)						
(1) Frontal Bilateral	1961 (125,504)	<0.001	−7	2	32	Middle Frontal Gyrus (MidFG), LR Precentral Gyrus (PreCG),LR Superior Frontal Gyrus (SFG), LR Supplementary Motor Cortex (SMA), LR Thalamus, LR Putamen, LR
(2) Parietal Bilateral	665 (42,560)	<0.001	−14	−52	52	Precuneous Cortex Superior Parietal Lobule (SPL), LR Lateral Occipital Cortex, superior division (sLOC), LR Postcentral Gyrus (PostCG), L Supramarginal Gyrus, posterior division (pSMG), L
(3) Occipito-temporal Left	127 (8128)	<0.001	−42	−68	19	Lateral Occipital Cortex, superior division (sLOC), L Middle Temporal Gyrus, temporooccipital part (toMTG), L
(4) Occipital Right	59 (3776)	0.002	40	−69	25	Lateral Occipital Cortex, superior division (sLOC), R
(5) Parietal Right	33 (2112)	0.020	36	−39	39	Supramarginal Gyrus, posterior division (pSMG), R Superior Parietal Lobule (SPL), R
Rest (Baseline) > Imagery						
(1) Right Operculum	658 (28,032)	<0.001	48	−17	8	Central Opercular Cortex (CO), R Parietal Operculum Cortex (PO), R Insular Cortex (IC), R Middle Temporal Gyrus, posterior division (pMTG), R Superior Temporal Gyrus, posterior division (pSTG), R Heschl’s Gyrus (HG), R Planum Temporale (PT), R
(2) Left Operculum	354 (42,112)	<0.001	−49	−23	7	Central Opercular Cortex (CO), L Parietal Operculum Cortex (PO), L Insular Cortex (IC), L Middle Temporal Gyrus, posterior division (pMTG), L Superior Temporal Gyrus, posterior division (pSTG), L Heschl’s Gyrus (HG), L Planum Temporale (PT), L Planum OPerculum (PO), L
(3) Occipital Bilateral	438 (22,656)	<0.001	5	−79	14	Lateral Occipital Cortex, superior division (sLOC), LR Lateral Occipital Cortex, inferior division (iLOC), LR Cuneal Cortex, LR Occipital Pole (OP), LR Lingual Gyrus (LG), LR
(4) Medial Frontal	281 (17,984)	<0.001	7	48	16	Paracingulate Gyrus (PaCiG), LR Cingulate Gyrus, anterior division (AC) Frontal Pole (FP), R Superior Frontal Gyrus (SFG), LR
(5) Left Angular Gyrus	25 (1600)	0.028	−53	−54	31	Angular Gyrus (AG), L
(6) Right Angular Gyrus	24 (1536)	0.028	55	−54	35	Angular Gyrus (AG), R
(7) Posterior Cingulate	36 (2304)	0.014	4	−19	46	Cingulate Gyrus, posterior division (PC) Precentral Gyrus (PreCG), R
(8) Right Postcentral	32 (2048)	0.017	49	−20	52	Postcentral Gyrus (PostCG), R
(9) Left Postcentral	24 (1536)	0.028	−44	−22	61	Postcentral Gyrus (PostCG), L

^1^ For each cluster, only the anatomical labels covering more than 5% of cluster volume are presented; voxel size is 4 mm isotropic.

**Table 2:** Clusters of activation specific to imaginary tennis and house navigation tasks (compared to the other task) across both vantage points in all participants.

Cluster	Volume, Voxels (mm^3^)	*p*_FDR_	MNI Coordinates (Center of Mass)			Region Labels ^1^
			x	y	z	
Tennis > Navigation						
(1) SMA	56 (3584)	0.001	−9	−7	59	Supplementary Motor Cortex (SMA), LR Precentral Gyrus (PreCG), L
(2) Right Supramarginal Gyrus	51 (3264)	0.001	61	−34	27	Supramarginal Gyrus, posterior division (pSMG), R Supramarginal Gyrus, anterior division (aSMG), R Parietal Operculum Cortex (PO), R
(3) Left Superior Parietal	32 (2048)	0.008	−35	−43	60	Superior Parietal Lobule (SPL), L
Navigation > Tennis						
(1) Left Para-hippocampal	51 (3264)	0.003	−26	−41	−10	Parahippocampal Gyrus, posterior division (pPaHC), L Lingual Gyrus (LG), L Hippocampus L
(2) Right Para-hippocampal	36 (2304)	0.010	27	−38	−10	Parahippocampal Gyrus, posterior division (pPaHC), R Lingual Gyrus (LG), R Hippocampus R
(3) Bilateral Occipito-temporal	766 (49,024)	<0.001	−4	−62	34	Precuneous Lateral Occipital Cortex, superior division (sLOC), LR Cingulate Gyrus, posterior division (PC)
(4) Right Lateral Occipital Cortex	74 (4736)	0.001	38	−76	28	Lateral Occipital Cortex, superior division (sLOC), R
(5) Left Frontal	66 (4224)	0.001	−28	13	50	Middle Frontal Gyrus (MFG), L Superior Frontal Gyrus (SFG), L
(6) Right Frontal	22 (1408)	0.035	26	9	53	Middle Frontal Gyrus (MFG), R Superior Frontal Gyrus (SFG), R

^1^ For each cluster, only the anatomical labels covering more than 5% of cluster volume are presented; voxel size is 4 mm isotropic.

**Table 3:** Results of the NBS connectivity analysis (mass/intensity) for the first-person vs. third-person perspective contrast in both the imaginary tennis and house navigation tasks considered together.

Analysis Unit	Mass	t(24)	p-unc.	*p*_FDR_
Network 1/2	108.87		0.022553	0.045
Connection ICCl—pSTGl		4.76	0.000077	0.421
Connection pMTGl—ICCl		4.19	0.000327	0.800
Connection pMTGl—SCCr		3.78	0.000926	0.999

ICCl: the intracalcarine cortex, left; pSTGl: the superior temporal gyrus, posterior division, left; pMTGl: the middle temporal gyrus, posterior division, right.

## Data Availability

The data reported in the paper are available in the Openneuro repository: https://doi.org/10.18112/openneuro.ds006077.v1.0.0 (accessed on 2 April 2025).

## References

[1] Tversky B., Hard B.M. (2009). Embodied and Disembodied Cognition: Spatial Perspective-Taking. Cognition.

[2] Piaget J. (1954). The Construction of Reality in the Child.

[3] Nigro G., Neisser U. (1983). Point of View in Personal Memories. Cogn. Psychol..

[4] Libby L.K., Eibach R.P. (2011). Visual Perspective in Mental Imagery. Advances in Experimental Social Psychology.

[5] Kosslyn S.M., Ganis G., Thompson W.L. (2001). Neural Foundations of Imagery. Nat. Rev. Neurosci..

[6] Zacks J.M., Mires J., Tversky B., Hazeltine E. (2000). Mental spatial transformations of objects and perspective. Spat. Cogn. Comput..

[7] Hong J.P. (2024). The Influence of Visual Perspective on the Cognitive Effort Required for Mental Representation. Ph.D. Thesis.

[8] Hegarty M. (2004). A Dissociation between Mental Rotation and Perspective-Taking Spatial Abilities. Intelligence.

[9] Galvan Debarba H., Bovet S., Salomon R., Blanke O., Herbelin B., Boulic R. (2017). Characterizing First and Third Person Viewpoints and Their Alternation for Embodied Interaction in Virtual Reality. PLoS ONE.

[10] Jackson P.L., Meltzoff A.N., Decety J. (2006). Neural Circuits Involved in Imitation and Perspective-Taking. NeuroImage.

[11] Higuchi T., Nagami T., Nakata H., Watanabe M., Isaka T., Kanosue K. (2016). Contribution of Visual Information about Ball Trajectory to Baseball Hitting Accuracy. PLoS ONE.

[12] Avraamides M.N., Klatzky R.L., Loomis J.M., Golledge R.G. (2004). Use of Cognitive Versus Perceptual Heading During Imagined Locomotion Depends on the Response Mode. Psychol. Sci..

[13] Burgess N. (2006). Spatial Memory: How Egocentric and Allocentric Combine. Trends Cogn. Sci..

[14] Klatzky R.L., Freksa C., Habel C., Wender K.F. (1998). Allocentric and Egocentric Spatial Representations: Definitions, Distinctions, and Interconnections. Spatial Cognition.

[15] Zaehle T., Jordan K., Wüstenberg T., Baudewig J., Dechent P., Mast F.W. (2007). The Neural Basis of the Egocentric and Allocentric Spatial Frame of Reference. Brain Res..

[16] Kozhevnikov M., Hegarty M. (2001). A Dissociation between Object Manipulation Spatial Ability and Spatial Orientation Ability. Mem. Cogn..

[17] Vogeley K., Fink G.R. (2003). Neural Correlates of the First-Person-Perspective. Trends Cogn. Sci..

[18] Leng X., Zhu W., Mayer R.E., Wang F. (2024). The Viewing Perspective Effect in Learning from Instructional Videos: A Replication and Neuroimaging Extension. Learn. Instr..

[19] Pavone E.F., Tieri G., Rizza G., Tidoni E., Grisoni L., Aglioti S.M. (2016). Embodying Others in Immersive Virtual Reality: Electro-Cortical Signatures of Monitoring the Errors in the Actions of an Avatar Seen from a First-Person Perspective. J. Neurosci..

[20] Cammisuli D.M., Castelnuovo G. (2023). Neuroscience-Based Psychotherapy: A Position Paper. Front. Psychol..

[21] Ruby P., Decety J. (2001). Effect of Subjective Perspective Taking during Simulation of Action: A PET Investigation of Agency. Nat. Neurosci..

[22] Tomasino B., Werner C.J., Weiss P.H., Fink G.R. (2007). Stimulus Properties Matter More than Perspective: An fMRI Study of Mental Imagery and Silent Reading of Action Phrases. NeuroImage.

[23] Arzy S., Thut G., Mohr C., Michel C.M., Blanke O. (2006). Neural Basis of Embodiment: Distinct Contributions of Temporoparietal Junction and Extrastriate Body Area. J. Neurosci..

[24] Blanke O., Mohr C., Michel C.M., Pascual-Leone A., Brugger P., Seeck M., Landis T., Thut G. (2005). Linking Out-of-Body Experience and Self Processing to Mental Own-Body Imagery at the Temporoparietal Junction. J. Neurosci..

[25] Smith A.M., Messier C. (2014). Voluntary Out-of-Body Experience: An fMRI Study. Front. Hum. Neurosci..

[26] Gauthier B., Bréchet L., Lance F., Mange R., Herbelin B., Faivre N., Bolton T.A.W., Ville D.V.D., Blanke O. (2020). First-Person Body View Modulates the Neural Substrates of Episodic Memory and Autonoetic Consciousness: A Functional Connectivity Study. NeuroImage.

[27] Grol M., Vingerhoets G., De Raedt R. (2017). Mental Imagery of Positive and Neutral Memories: A fMRI Study Comparing Field Perspective Imagery to Observer Perspective Imagery. Brain Cogn..

[28] Eich E., Nelson A.L., Leghari M.A., Handy T.C. (2009). Neural Systems Mediating Field and Observer Memories. Neuropsychologia.

[29] St. Jacques P.L. (2019). A New Perspective on Visual Perspective in Memory. Curr. Dir. Psychol. Sci..

[30] Owen A.M., Coleman M.R., Boly M., Davis M.H., Laureys S., Pickard J.D. (2006). Detecting Awareness in the Vegetative State. Science.

[31] Oldfield R.C. (1971). The Assessment and Analysis of Handedness: The Edinburgh Inventory. Neuropsychologia.

[32] Fernández-Espejo D., Norton L., Owen A.M. (2014). The Clinical Utility of fMRI for Identifying Covert Awareness in the Vegetative State: A Comparison of Sensitivity between 3T and 1.5T. PLoS ONE.

[33] Desikan R.S., Ségonne F., Fischl B., Quinn B.T., Dickerson B.C., Blacker D., Buckner R.L., Dale A.M., Maguire R.P., Hyman B.T. (2006). An Automated Labeling System for Subdividing the Human Cerebral Cortex on MRI Scans into Gyral Based Regions of Interest. NeuroImage.

[34] Nieto-Castanon A. (2020). Handbook of Functional Connectivity Magnetic Resonance Imaging Methods in CONN.

[35] Whitfield-Gabrieli S., Nieto-Castanon A., Ghosh S. (2011). Artifact Detection Tools (ART).

[36] Behzadi Y., Restom K., Liau J., Liu T.T. (2007). A Component Based Noise Correction Method (CompCor) for BOLD and Perfusion Based fMRI. NeuroImage.

[37] Martuzzi R., Ramani R., Qiu M., Shen X., Papademetris X., Constable R.T. (2011). A Whole-Brain Voxel Based Measure of Intrinsic Connectivity Contrast Reveals Local Changes in Tissue Connectivity with Anesthetic without a Priori Assumptions on Thresholds or Regions of Interest. NeuroImage.

[38] Chumbley J., Worsley K., Flandin G., Friston K. (2010). Topological FDR for Neuroimaging. NeuroImage.

[39] Bullmore E.T., Suckling J., Overmeyer S., Rabe-Hesketh S., Taylor E., Brammer M.J. (1999). Global, Voxel, and Cluster Tests, by Theory and Permutation, for a Difference between Two Groups of Structural MR Images of the Brain. IEEE Trans. Med. Imaging.

[40] Zalesky A., Fornito A., Bullmore E.T. (2010). Network-Based Statistic: Identifying Differences in Brain Networks. NeuroImage.

[41] Spagna A., Hajhajate D., Liu J., Bartolomeo P. (2021). Visual Mental Imagery Engages the Left Fusiform Gyrus, but Not the Early Visual Cortex: A Meta-Analysis of Neuroimaging Evidence. Neurosci. Biobehav. Rev..

[42] Dijkstra N. (2024). Uncovering the Role of the Early Visual Cortex in Visual Mental Imagery. Vision.

[43] Hoppe M., Baumann A., Tamunjoh P.C., Machulla T.-K., Woźniak P.W., Schmidt A., Welsch R. (2022). There Is No First- or Third-Person View in Virtual Reality: Understanding the Perspective Continuum. Proceedings of the CHI Conference on Human Factors in Computing Systems.

[44] Aïte A., Berthoz A., Vidal J., Roëll M., Zaoui M., Houdé O., Borst G. (2016). Taking a Third-Person Perspective Requires Inhibitory Control: Evidence from a Developmental Negative Priming Study. Child Dev..

[45] Guterstam A., Larsson D.E.O., Szczotka J., Ehrsson H.H. (2020). Duplication of the Bodily Self: A Perceptual Illusion of Dual Full-Body Ownership and Dual Self-Location. R. Soc. Open Sci..

[46] Sutton J. (2012). Memory Before the Game: Switching Perspectives in Imagining and Remembering Sport and Movement. J. Ment. Imag..

[47] Kinley I., Porteous M., Levy Y., Becker S. (2021). Visual Perspective as a Two-Dimensional Construct in Episodic Future Thought. Conscious. Cogn..

[48] Davey J., Thompson H.E., Hallam G., Karapanagiotidis T., Murphy C., De Caso I., Krieger-Redwood K., Bernhardt B.C., Smallwood J., Jefferies E. (2016). Exploring the Role of the Posterior Middle Temporal Gyrus in Semantic Cognition: Integration of Anterior Temporal Lobe with Executive Processes. NeuroImage.

[49] Phylactou P., Traikapi A., Papadatou-Pastou M., Konstantinou N. (2022). Sensory Recruitment in Visual Short-Term Memory: A Systematic Review and Meta-Analysis of Sensory Visual Cortex Interference Using Transcranial Magnetic Stimulation. Psychon. Bull. Rev..

[50] Kosslyn S.M. (1980). Image and Mind.

